# Public Pharmacists' Association

**Published:** 1920-03-13

**Authors:** 


### Public Pharmacists' Association.

At a recent meeting held at St. Bride Institute Mr. J. L. P. Hollingworth, M.P.S., F.C.S., Victoria Hospital, Chelsea, presided over a gathering of pharmacists attached to voluntary hospitals and other institutions. The evening was devoted to "Short Papers by Members," and the following contributions were read: "The Culture of Pharmacy," by J. L. P. Hollingworth; "Aids to Dispensing," by A. H. Jenkin; "My War Experiences," by F. E. Bullen, late sergeant, R.A.M.C.

Mr. Hollingworth advocated education on generous lines for those entering pharmacy, and suggested that the time was ripe for instituting a preliminary examination to admit students to the medical, dental, and pharmaceutical professions on equal terms.

Mr. Jenkin, who is a member of the Pharmaceutical Council for Great Britain, in his paper, gave some valuable "tips" on the best way to expedite dispensary work, by the method of employing simple but effective mechanical helps, such as syphons, patent label slots, rapid filters, etc. Members were greatly interested in this practical paper on things pertaining to the everyday work of the institutional pharmacist, and the discussion led various members to explain appliances which they had found useful in their work.

Mr. Bullen's paper dealt with experiences in the R.A.M.C., both at home and abroad. He deplored the fact that pharmacists were, in many cases, found no more useful work than that of cleaning windows! In his own case he acted as lance-corporal under a sergeant who had in civil life been a moulder's labourer. He hoped that the representations made to the War Office by the Pharmaceutical Society would result in an Army Pharmaceutical Service—on the lines of that adopted by France and other of our Allies. Mr. Bullen described in vivid language the places at which he had been stationed; these included Basra and Amarah, in Mesopotamia.

The meeting decided to vote 1*l*. 5*s*. to the Benevolent Fund of the Pharmaceutical Society of Great Britain, which does useful work in assisting chemists and their dependants who have fallen on hard days. It was also decided to invite the Benevolent Fund Committee to forward a collecting-box for use at future meetings.

